# Acceptability and Fidelity of a Cognitive Rehabilitation Intervention During and After Intensive Care: A Feasibility Evaluation

**DOI:** 10.1111/nicc.70544

**Published:** 2026-06-16

**Authors:** Katrine Astrup, Anna Holm, Helene Korvenius Nedergaard, Nanna Rolving, Pia Dreyer

**Affiliations:** ^1^ Department of Intensive Care Aarhus University Hospital Aarhus Denmark; ^2^ Department of Physiotherapy and Occupational Therapy Aarhus University Hospital Aarhus Denmark; ^3^ Department of Public Health Aarhus University Aarhus Denmark; ^4^ Department of Anaesthesiology and Intensive Care University Hospital of Southern Denmark, Lillebaelt Hospital Kolding Kolding Denmark; ^5^ Department of Regional Health Research University of Southern Denmark Odense Denmark

**Keywords:** acceptability, cognitive rehabilitation, critical care, feasibility study, intensive care unit, intervention fidelity

## Abstract

**Background:**

Cognitive impairment is common after critical illness, yet structured cognitive rehabilitation is rarely integrated into routine intensive care practice. ICU CogHab is a stakeholder‐ and theory‐informed intervention comprising two components: Mindfulness and Brain Training, developed to support recovery from intensive care to 6‐month follow‐up.

**Aim:**

To evaluate the feasibility of the ICU CogHab intervention, focussing on acceptability and fidelity among ICU patients and nurses.

**Study Design:**

A multi‐method feasibility evaluation was conducted alongside a pragmatic, five‐arm randomised controlled feasibility trial in four Danish ICUs. Data were collected using patient and nurse surveys and semi‐structured interviews. Acceptability was explored in terms of three domains: affective attitude, burden and perceived effectiveness. Fidelity was assessed in relation to adherence and dose, with participant responsiveness and quality of delivery examined within adherence. Quantitative data were analysed descriptively, and qualitative data were analysed using deductive content analysis.

**Results:**

Thirty‐one patients contributed with survey data and nine participated in interviews. In addition, 53 nurses contributed with 194 session‐based surveys and seven interviews, informing evaluation of delivery during ICU admission. Both components were generally perceived as relevant and appropriate by patients and nurses. Patients described the components as meaningful and supportive. Fidelity varied across settings and over time, with adherence and dose influenced by clinical condition, fatigue, patient readiness and competing rehabilitation demands, meaning that intervention use often required adaptation in timing and duration across recovery phases. Nurses reported contextual barriers to delivery, including high workload and competing clinical priorities.

**Conclusion:**

The ICU CogHab intervention components demonstrated overall acceptability across ICU and post‐discharge phases. Variation in fidelity reflected the patient condition, clinical context and competing demands, providing direction for further refinement before larger‐scale evaluation.

**Relevance to Clinical Practice:**

Cognitive rehabilitation may be acceptable and feasible to introduce within intensive care pathways but delivery may require flexible timing, integration into routine care and support across the ICU‐to‐home transition.

## Impact Statements

- What is known about the topic?
  ○ Cognitive impairment is common after critical illness and may contribute to persistent difficulties in everyday functioning and participation.
  ○ Despite this, structured cognitive rehabilitation is not routinely integrated into intensive care pathways.
  ○ Emerging evidence suggests that cognitive interventions may be feasible, but there is limited knowledge regarding their acceptability and how they are delivered and used across the ICU‐to‐home recovery trajectory.
- What this paper adds?
  ○ This study demonstrates that two cognitive rehabilitation components, Mindfulness and Brain Training, were perceived as acceptable and meaningful by both patients and nurses during ICU admission and after discharge.
  ○ However, fidelity varied across recovery stages and care settings and was influenced by patient readiness, clinical context and available support, affecting both adherence and dose.
  ○ The findings highlight the importance of flexible delivery, timing and sustained support across the ICU‐to‐home transition to support consistent engagement over time.

**Abbreviations**
COREQ: Consolidated Criteria for Reporting Qualitative Research
CROSS: Consensus‐Based Checklist for Reporting of Survey Studies
GDPR: General Data Protection Regulation
ICU: Intensive Care Unit
IQR: Interquartile Range
MRC: Medical Research Council
OSF: Open Science Framework
QR (code): Quick Response
REDCap: Research Electronic Data Capture
SD: Standard Deviation
TFA: Theoretical Framework of Acceptability
TIDieR: Template for Intervention Description and Replication

## 1 Introduction

Post‐Intensive Care Syndrome (PICS) has been increasingly recognised over the past two decades and is defined as *new or worsening impairments in physical, cognitive or mental health status arising after critical illness and persisting beyond hospital discharge* [1]. Among these domains, cognitive impairment is one of the most persistent and under‐recognised sequelae of critical illness [2, 3]. Many ICU survivors experience deficits in attention, memory and executive functioning months or years after discharge [4, 5], which may substantially affect everyday life, social participation, return to work and long‐term healthcare utilisation [3, 6].

## 2 Background

Research highlights the lived consequences of post‐ICU cognitive impairment, with survivors describing memory problems, challenges in managing daily activities and the feeling of insufficient support during recovery [7, 8]. At the same time, nurses in intensive care report uncertainty and a lack of structured, evidence‐based approaches to address cognitive rehabilitation in the early phases of critical illness [9]. Together, these findings indicate a gap in care across the transition from ICU admission to the ward and into post‐discharge recovery.

Emerging evidence from cognitive rehabilitation research within critical illness, including the RETURN I and II trials, suggests that structured cognitive interventions may support recovery when delivered early and consistently [10, 11]. However, evidence remains limited regarding how such interventions are perceived, delivered and sustained across different phases of recovery.

Drawing on principles from neurorehabilitation and mindfulness‐based approaches, strategies targeting structured cognitive stimulation, attentional control and emotional regulation may be particularly relevant for ICU survivors with varying needs and fluctuating capacity [12, 13, 14].

The ICU CogHab intervention was developed to address this gap, and it comprises two intervention components, Mindfulness and Brain Training, delivered from ICU admission to six months after ICU discharge (Astrup et al. under review). Within the feasibility study, patients were randomised to Mindfulness, Brain Training or usual care. Evaluation of how these components are perceived, delivered and used in practice is therefore needed.

## 3 Aim

The aim of this study was to evaluate the feasibility of the ICU CogHab intervention, with particular focus on acceptability and fidelity among ICU patients and nurses, to inform further intervention refinement and future evaluation.

## 4 Methods

### 4.1 Study Design

This feasibility study reports the acceptability and fidelity of a cognitive rehabilitation intervention (ICU CogHab), evaluated within a five‐arm randomised controlled feasibility trial with 6‐month follow‐up. The trial comprised four intervention groups (two with post‐ICU support and two without) and a usual care group. Evaluation of study design feasibility outcomes, including recruitment, retention and trial procedures, has been reported separately [15].

Guided by the Medical Research Council (MRC) framework for complex interventions [16], the present multi‐method feasibility evaluation combined quantitative survey data with qualitative interviews to explore experiences of the intervention components in terms of relevance, perceived burden and effect and contextual influences on fidelity (i.e., delivery and engagement).

A 12‐month inclusion period was selected to generate sufficient quantitative and qualitative data for feasibility evaluation while accounting for expected attrition. Quantitative survey components are reported in accordance with the Consensus‐Based Checklist for Reporting of Survey Studies (CROSS) [17], and qualitative interviews followed the Consolidated Criteria for Reporting Qualitative Research (COREQ) [18].

#### 4.1.1 Intervention

The ICU CogHab intervention comprised two structured cognitive rehabilitation components: Mindfulness and Brain Training. Patients were allocated to either: (1) Mindfulness with ICU support only, (2) Brain Training with ICU support only, (3) Mindfulness with ICU and post‐ICU support, (4) Brain Training with ICU and post‐ICU support or (5) usual care. Both components were delivered using dedicated intervention boxes containing all relevant materials and were intended to accompany patients from ICU admission until 6 months post‐discharge. ICU nurses (hereafter referred to as nurses) were trained to initiate and support intervention use during admission as part of routine care. Patients received written instructions, and relatives were also encouraged to assist and support engagement when feasible. The Mindfulness component consisted of brief, audio‐guided exercises delivered via QR codes to support attentional and emotional regulation. The Brain Training component included graded cognitive exercises targeting attention, memory and executive function, delivered through a structured activity book and simple supporting tools. The intervention was designed to be flexible, allowing adaptation according to individual capacity and preferences.

Two intervention groups additionally received structured post‐ICU support for up to 6 months following discharge, consisting of planned follow‐up contacts (e.g., in‐person visits, telephone calls, or text messages) to encourage continued engagement. Detailed descriptions of intervention development, content and delivery are reported elsewhere (Astrup et al. under review) and in accordance with the template for intervention description and replication (TIDieR) checklist [19].

### 4.2 Setting and Participants

The study was conducted in four medical and surgical ICUs across two Danish university hospitals, including approximately 30 ICU beds in total, providing high‐dependency care, including invasive mechanical ventilation and continuous haemodynamic monitoring, with predominantly 1:1 nurse‐to‐patient staffing. Daily patient care was guided by principles aligned with national and international recommendations [20, 21], including light or minimal sedation enabling many patients to remain awake and participate in care, routine delirium assessment, early mobilisation and supportive strategies such as sleep promotion, reorientation, communication support and active involvement of relatives. These practices were considered important contextual conditions underpinning the intervention, as they were expected to support attentional engagement and readiness to participate in cognitive rehabilitation activities during ICU admission. However, structured cognitive rehabilitation was not routinely integrated into usual care.

In the parent feasibility trial, patients were eligible if they were expected to require ICU admission for more than 24 h, were able to communicate in Danish and were awake and without evidence of delirium at inclusion according to routine clinical assessment. Across the participating ICUs, 115 patients met the eligibility criteria, of whom 83 were enrolled in the feasibility trial [15].

All consenting patients randomised to an intervention group were asked to complete intervention evaluation surveys following ICU discharge and through 6‐month follow‐up. Patients eligible for qualitative interviews were those who had used the intervention partially or until follow‐up and had consented to interview participation. Patients allocated to usual care were not interviewed.

Across the four ICUs, nurses delivered the intervention components as part of routine care. When caring for patients allocated to an intervention group, they were instructed, where feasible, to support use of the materials and complete brief surveys related to the intervention delivery and use. Nurses with direct delivery experience were subsequently invited to participate in qualitative interviews.

### 4.3 Feasibility Outcomes

The feasibility evaluation focused on two primary domains: acceptability and fidelity. Acceptability was informed by three domains from the Theoretical Framework of Acceptability (TFA) (affective attitude, burden, perceived effectiveness) [22], and fidelity was guided by Carroll et al. [23] and operationalised through measures of adherence and dose. Indicators related to quality of delivery and participant responsiveness were examined within the adherence domain, with patient‐reported data reflecting intervention use and engagement, and nurse‐reported data reflecting delivery and practical implementation. Quantitative survey data and qualitative interviews were used to assess predefined domains within these frameworks. An overview of domains, definitions and data sources is provided in Table 1.

**TABLE 1:** Data collection in relation to the acceptability and fidelity domains, including definitions and measurement methods.

Domain	Definition	Quantitative assessment	Qualitative assessment	Data source
Acceptability				
Affective Attitude	How participants feel about the intervention	Likert‐scale survey items	Interviews on relevance and overall experience; free‐text comments	Patients, nurses
Burden	Perceived amount of effort required to participate in or deliver the intervention	Likert‐scale survey items	Interviews on burden, practical challenges, barriers and facilitators; free‐text comments	Patients, nurses
Perceived effectiveness	The extent to which participants believe the intervention is able to achieve its intended aims (e.g., supporting cognitive recovery)	Not assessed quantitatively	Interviews on perceived benefits and impact; free‐text comments	Patients, nurses
Fidelity				
Adherence	Completion of planned components			
	How those responsible for delivering (nurses) and using (patients) the intervention adhere to the intervention as intended	Survey: component checklist to tick‐off	Interviews on intervention use; free‐text comments	Patients, nurses
	Indicators of delivery quality			
	How intervention is delivered in an appropriate way to achieve what was intended (including competence and consistency in delivering the intervention)	Not assessed quantitatively	Interviews on delivery quality; free‐text comments	
	Indicators of participant engagement			
	Level of engagement and receptiveness of those who deliver (nurses) and use (patients) the intervention	Not assessed quantitatively	Interviews on engagement; free‐text comments	
Exposure/dose	Amount of intervention delivered/received	Survey: number of sessions (tick‐off), duration of use	Interviews on frequency and duration of use; free‐text comments	Patients, nurses

*Note:* Monitoring of survey completion was conducted across all domains. Free‐text comments include written survey responses.

### 4.4 Data Collection

#### 4.4.1 Quantitative Data Collection

Quantitative data were collected using two surveys: an ICU‐based survey completed by the nurses after each intervention session during ICU admission, and a follow‐up survey completed weekly by patients after ICU discharge until 6‐month follow‐up (distributed every Sunday for 26 weeks).

Survey instruments were developed specifically for this study and underwent face validation by the research group and experienced ICU nurses (*n* = 3) to ensure clarity and alignment with predefined feasibility domains. Items included multiple‐choice questions and 5‐point Likert scale ratings (1 = strongly disagree to 5 = strongly agree) [24]. Survey items were designed to capture key indicators of acceptability (e.g., perceived relevance and appropriateness in relation to patients' energy level) and fidelity (e.g., use, frequency, duration, content chosen and level of support). Given the clinical condition of ICU patients and the longitudinal follow‐up design, survey length was intentionally kept brief to minimise burden and support completion. Both surveys also included optional free‐text fields allowing patients and nurses to provide additional comments on intervention use and experiences. During ICU admission, the ICU survey captured both nurse‐reported information on intervention delivery and patient‐reported responses to the intervention, which were obtained and recorded by nurses directly in the survey system. The survey was accessed via QR code on a mobile phone or a designated tablet. Following ICU discharge, patients received brief weekly surveys via text messages, which were completed with optional support from relatives. All survey data were collected using the Research Electronic Data Capture (REDCap) system [25].

#### 4.4.2 Qualitative Data Collection

Qualitative data included semi‐structured interviews with patients and nurses, supplemented by free‐text survey responses. Patient interviews were conducted around 6‐month follow‐up, either face‐to‐face in the hospital or by telephone according to patients' preferences. Nurse interviews were conducted following study completion. The interview guides were developed to explore experiences related to acceptability of the intervention components and fidelity in terms of contextual factors influencing delivery and engagement. Interview guides are provided in Supplementary Files A, B and C. All interviews were audio‐recorded and subsequently transcribed subsequently. Audio recordings were transcribed using an AI‐assisted transcription tool (Whisper Transcription, Aarhus University). All transcripts were manually checked against the recordings and anonymised prior to analysis. Qualitative coding and data organisation were performed in Microsoft Word.

### 4.5 Data Analysis

#### 4.5.1 Quantitative Analysis

Quantitative survey data were analysed descriptively in accordance with the study's aims. Continuous variables are reported as means with SD when normally distributed, or as medians with interquartile ranges (IQR) otherwise. Categorical variables are presented as frequencies and percentages. Surveys were analysed at item and domain level according to the pre‐defined feasibility outcomes and presented separately for patients and nurses. Where relevant, proportions indicating agreement were reported to support interpretation of feasibility signals, defined as indicative patterns of acceptability and intervention use rather than formal hypothesis testing. All analyses were conducted using Stata version 19.5 (StataCorp, College Station, TX, USA).

#### 4.5.2 Qualitative Analysis

Qualitative data, including interview transcripts and free‐text survey responses, were analysed collectively using deductive content analysis informed by Elo and Kyngäs [26].

An analysis matrix was developed a priori based on the TFA framework [22] and the fidelity framework [23]. All data were read repeatedly to achieve familiarisation and subsequently organised and coded into predefined categories reflecting acceptability (affective attitude, burden, perceived effectiveness) and fidelity (adherence, exposure/dose). Indicators related to quality of delivery and participant responsiveness were examined within the adherence domain. Coding was conducted by the primary author with regular discussions with members of the research group to ensure consistency of interpretation. Findings were summarised within each domain and supported by representative quotations.

### 4.6 Ethical Considerations

The study was conducted in accordance with the Declaration of Helsinki [27] and approved March 5th, 2024, by the local Research Ethics Committee (record no. 1‐10‐72‐137‐23). The study was prospectively registered in the Open Science Framework (osf.io/b57fv/overview). Written informed consent was obtained from all included patients prior to inclusion. Nurses provided informed consent for participation in interviews, and survey data were collected using pseudonymised identifiers. Data were handled in compliance with the General Data Protection Regulation (GDPR) and stored securely at a database hosted at Central Denmark Region with access restricted to members of the research group.

### 4.7 Disclosing the Use of Artificial Intelligence (AI)

During the preparation of the manuscript, the authors used OpenAI's ChatGPT (version 5.0, OpenAI, USA) for editorial support limited to language refinement and enhancement of clarity. No identifiable or sensitive personal data were entered in the system. Following use of this tool, the authors reviewed and edited the content as necessary and take full responsibility for the final manuscript.

## 5 Results and Findings

Eighty‐three patients were enrolled in the parent randomised feasibility trial and allocated across the five study groups. Detailed study flow and evaluation are reported in the parent feasibility trial publication [15]. The present feasibility evaluation included patients allocated to the four intervention groups. Of these, 31 completed at least one follow‐up survey and nine participated in interviews. In addition, 53 nurses completed 194 ICU registrations, and seven participated in interviews. Due to the relatively small numbers within individual intervention groups and participating ICUs, findings were interpreted as exploratory feasibility signals related to intervention acceptability, delivery and engagement across recovery phases rather than as formal subgroup comparisons. All data were collected between June 2024 and December 2025. Participant characteristics for survey and interview samples are presented in Table 2. Survey response counts varied across items due to item‐level missing responses. Patient interviews lasted 15–46 min and nurse interviews 13–26 min. No unintended events related to the study procedures were identified.

**TABLE 2:** Participant characteristics for surveys and interviews.

Survey participants (patients)	*n* = 31
Intervention type	
Mindfulness, *n* (%)	13 (42)
Brain Training, *n* (%)	18 (58)
Gender	
Female, *n* (%)	15 (48)
Male, *n* (%)	16 (52)
Age, years, mean (SD)	65 (10.6)
Civil status	
Living with spouse/other (e.g., children)	22 (71)
Living alone	8 (26)
Other/unknown	1 (3)
ICU length of stay, days, median (range)	6 (2–18)

Interview participants (patients)	*n* = 9
Intervention type	
Mindfulness, *n* (%)	4 (44)
Brain Training, *n* (%)	5 (56)
Gender	
Female, *n* (%)	6 (67)
Male, *n* (%)	3 (33)
Age, years, median (range)	60 (36–77)
Civil status	
Living with spouse/other (e.g., children)	8 (89)
Living alone	1 (11)
ICU length of stay, days, median (range)	6 (2–15)

Interview participants (nurses)	*n* = 7
Gender	
Female, *n* (%)	6 (86)
Male, *n* (%)	1 (14)
Age, years, median (range)	38 (27–57)
Clinical nursing experience, years, median (range)	9 (2–31)
Clinical ICU experience, years, median (range)	5 (2–25)
Critical care nursing degree, *n* (%)	
Yes	4 (57)
No	3 (43)

*Note:* No additional ICU nurse characteristics were collected for survey respondents, as survey data were pseudonymised.

### 5.1 Acceptability of the Intervention

Findings related to affective attitude, burden and perceived effectiveness are presented below.

#### 5.1.1 Quantitative Indicators of Acceptability

Nurse registrations during ICU admission indicated high perceived relevance and appropriateness of both intervention components in relation to the patients' energy level (Table 3).

**TABLE 3:** Nurse‐reported indicators of acceptability and fidelity during ICU admission.

Indicators	Mindfulness intervention	Brain training intervention
Fidelity indicators		
Use of the component, *n* (%)		
Yes	34 (40)	61 (55)
No	50 (60)	49 (45)
Time period of intervention use,^a^ *n* (%)	*n* = 34	*n* = 61
12:00–15:00 (early afternoon)	6 (18)	21 (34)
15:00–18:00 (late afternoon)	3 (9)	18 (30)
18:00–21:00 (evening)	12 (35)	13 (21)
Other	13 (38)	9 (15)
Time spent per session,^a^ min	*n* = 34	*n* = 61
Median (range)	15 (7–40)	20 (1–200)
Independence (completion of the intervention),^a^ *n* (%)	*n* = 34	*n* = 61
Independently	10 (26)	39 (51)
With support from the ICU nurse	21 (55)	19 (25)
With support from relative	1 (3)	13 (17)
With support from others (therapist, friend etc.)	6 (16)	5 (7)
Type of content used,^b^ *n*	*n* = 34	*n* = 61
Mindfulness—selected audio files		
Compassionate breath (male voice)	13	—
Compassionate breath (female voice)	4	—
Body scan	11	—
Comforting touch	4	—
Brain Training—activities		
Images and figures	—	17
Letters and words	—	13
Numbers and calculations	—	10
Creativity and patterns	—	3
Games and questions	—	5
Squeeze ball	—	36
Calander	—	6
Other	—	10
Acceptability indicators		
Perceived relevance	*n* = 31	*n* = 58
Median (IQR)	4 (4–4)	4 (4–5)
Agree or strongly agree, *n* (%)	24 (77)	47 (81)
Appropriateness in relation to patient's energy level	*n* = 31	*n* = 58
Median (IQR)	4 (3–4)	4 (4–5)
Agree or strongly agree, *n* (%)	23 (74)	48 (83)

*Note:* Data are presented as *n* (%) or median (range, IQR) across nurse registrations completed during ICU admission. Results (*n*) are based on individual registrations, and multiple registrations could be completed by the same nurse across patients and shifts. The number of responses per item is reported; missing data were not imputed.

^a^ Reported among registrations indicating delivery of the respective intervention component.

^b^ Multiple response options could be selected within the same registration; therefore, counts may exceed the number of registrations.

Among patient‐reported surveys following ICU discharge, both Mindfulness and Brain Training were rated as highly relevant (Affective attitude) (median 4 [IQR 4–4] for Mindfulness and 4 [IQR 3–4] for Brain Training) and appropriate to participants' energy level (Burden) (see Table 4).

**TABLE 4:** Patient‐reported indicators of acceptability and fidelity across repeated weekly survey responses from ICU discharge until 6‐month follow‐up.

Indicators	Mindfulness intervention	Brain training intervention
Fidelity indicators		
Use of the component, *n* (%)		
Yes	52 (48)	74 (56)
No	57 (52)	58 (44)
Frequency of use per week,^a^ *n* (%)	*n* = 52	*n* = 74
1–2 times	21 (40)	38 (53)
3–4 times	26 (50)	24 (34)
> 4 times	5 (10)	9 (13)
Time spent per session^a^	*n* = 50	*n* = 66
Minutes, median (range)	20 (10–60)	20 (5–120)
Independence (completion of the intervention),^a^ *n* (%)	*n* = 52	*n* = 74
Independently	50 (96)	58 (78)
With support from relative	1 (2)	11 (15)
With support from others (therapist, nurse, friend etc.)	1 (2)	5 (7)
Type of content used,^b^ *n*	*n* = 52	*n* = 74
Mindfulness – selected audio files		
Compassionate breath (male voice)	12	—
Compassionate breath (female voice)	18	—
Body scan	21	—
Comforting touch	10	—
Brain training – activities		
Images and figures	—	19
Letters and words	—	21
Numbers and calculations	—	22
Creativity and patterns	—	18
Games and questions	—	9
Squeeze ball	—	28
Calandar	—	30
Other	—	16
Acceptability indicators		
Perceived relevance	*n* = 49	*n* = 73
Median (IQR)	4 (4–4)	4 (4–4)
Agree or strongly agree, *n* (%)	38 (78)	62 (85)
Appropriateness in relation to patient's energy level	*n* = 47	*n* = 71
Median (IQR)	4 (4–4)	4 (3–4)
Agree or strongly agree, *n* (%)	38 (81)	53(75)

*Note:* Data are presented as *n* (%) or median (range or IQR) across completed weekly patient surveys during the post‐ICU follow‐up period. Results are based on individual survey responses, meaning that each patient could contribute multiple responses over time. The number of responses per item is reported; missing data were not imputed.

^a^ Reported among surveys indicating use of the respective intervention component.

^b^ Multiple response options could be selected within the same survey; therefore, counts may exceed the number of surveys.

#### 5.1.2 Qualitative Indicators of Acceptability

##### 5.1.2.1 Affective Attitude

Patients and nurses described predominantly positive emotional responses to engaging with the ICU CogHab components. Several patients initially expressed hesitation, scepticism or feelings of exhaustion when first introduced to the intervention. However, these reactions often shifted once they began engaging with the activities. As one patient reflected: ‘At first, when you presented it [Brain Training], I thought no… but then I realised it made sense’ (P1). Initial hesitation appeared to reflect early recovery‐phase challenges, including fatigue and reduced cognitive capacity, rather than negative appraisal of the intervention.

Mindfulness was described as calming, supportive and emotionally comforting: ‘It gives a sense of calm and detachment. That was what I needed’ (P4). One patient referred to it as ‘almost my lifeline’ (P5). These descriptions indicate that patients perceived mindfulness as providing emotional stability and reassurance during recovery. Brain Training was experienced as concrete, stimulating and motivating. Patients described it as engaging and purposeful, whereas nurses observed increased activation and motivation: ‘The Brain Training box worked very well, particularly in motivating patients’ (N1). Taken together, these findings indicate distinct patterns in how the two components were experienced, with Mindfulness associated with emotional regulation and Brain Training with cognitive activation and goal‐directed activity, a distinction also reflected in nurses' observations: ‘I definitely think the two boxes had distinct focuses’ (N1).

Across participants, engagement was associated with stimulation, motivation and a sense of meaningful activity during recovery. Overall, the components appeared distinct yet complementary, with experiences becoming more positive as familiarity with the intervention increased.

##### 5.1.2.2 Burden

Patients described both interventions as requiring energy and mental capacity that were not always available during recovery. Fluctuations in energy and fatigue influenced engagement over time, as reflected by one patient: ‘There have been periods where I haven't used it very much because I was too ill and didn't have the energy’ (P3). For several patients, even relatively simple activities could feel effortful in the early phase after discharge. The intervention components were generally acceptable but could be experienced as an additional demand alongside other rehabilitation tasks, medical appointments and symptom management. Mindfulness was sometimes associated with falling asleep due to exhaustion rather than disengagement, whereas Brain Training could be cognitively demanding when concentration was limited or other rehabilitation activities required prioritisation. Patients' open‐end survey comments similarly described illness, readmissions, competing life events and psychological vulnerability as factors increasing the perceived effort required to participate.

Among nurses, the intervention components were generally not described as particularly burdensome, but delivery required attention and prioritisation within a busy clinical environment: ‘Fitting the interventions into daytime shifts was challenging due to high activity levels and competing tasks’ (N1). Burden was therefore primarily related to contextual factors, including workload, patient fatigue and timing. Evening shifts were described as more suitable, though patient tiredness at that time could also limit capacity to participate. Patients' refusals due to tiredness were commonly reported in nurse registrations.

Overall, perceived burden was closely linked to fatigue, cognitive capacity and competing demands, indicating that engagement varied according to available energy and timing during recovery.

##### 5.1.2.3 Perceived Effectiveness

Many patients described the interventions as supporting cognitive and emotional aspects of recovery. One patient noted: ‘It has genuinely been something that helped me reset my mind a bit’ (P4), reflecting improved mental focus during rehabilitation.

Mindfulness was described as contributing to improved emotional regulation and psychological stability: ‘It gives a sense of calm and detachment. That was what I needed’ (P4), reflecting how these experiences supported the management of worry and mental overload. In contrast, Brain Training was more frequently associated with improved concentration, cognitive activation and everyday functioning: ‘The calendar became my way of prioritising’ (P8). Some patients described transferring strategies into daily routines and work‐related activities, suggesting perceived functional relevance beyond the immediate exercise context.

Nurses reported observable responses consistent with these experiences, including relaxation during Mindfulness and increased engagement during Brain Training: ‘The patient relaxed markedly and became calm’ *(Nurse survey comment)*. However, these responses depended on patient readiness and clinical timing.

Overall, perceived effectiveness was described in practical terms, including improved focus, emotional regulation and support during rehabilitation, while varying across recovery stages and individual capacity.

### 5.2 Intervention Fidelity

Findings related to adherence and dose of the ICU CogHab intervention are presented below.

#### 5.2.1 Quantitative Indicators of Fidelity

During ICU admission, delivery most commonly occurred between 12:00 and 21:00, with median session durations of approximately 15 min (Mindfulness) and 20 min (Brain Training) (Table 3). Brain Training often involved independent completion, whereas Mindfulness was more frequently nurse‐supported, with occasional involvement of relatives.

Among patient surveys reporting post‐discharge use, Mindfulness was most often completed three to four times per week and Brain Training one to two times per week (Table 4). Median session duration was approximately 20 min for both components. In contrast to the ICU phase, most Mindfulness sessions were completed independently (96%), whereas Brain Training more often involved support from relatives.

#### 5.2.2 Qualitative Indicators of Fidelity

##### 5.2.2.1 Adherence

Adherence was characterised by flexibility rather than uniformity across patients and settings. Activities were selected or postponed according to cognitive capacity, personal preference and stage of recovery. Simpler activities (e.g., squeeze ball, puzzle, painting exercises) were more commonly used during ICU admission, whereas more cognitively demanding tasks were perceived as more suitable later in recovery: ‘Some of the tasks [Brain Training] I could not do in the early phase… it was fine once I got home’ (P1). Several patients also described delayed initiation until they felt ready to engage: ‘It took some time before I got started with it [Mindfulness]… I simply wasn't ready’ (P5). Patients often repeated selected activities while avoiding others perceived as too demanding or lengthy (e.g., the body scan audio file). In some cases, activities were extended beyond the provided materials through use of alternative exercises or external resources, indicating continued engagement with the purpose of the intervention despite deviations from the original format.

Engagement was supported by perceived relevance, manageable format, integration into daily routines and support from relatives or the research group. Relatives were particularly important for Brain Training, facilitating use when independent engagement was challenging.

Patients allocated to intervention groups with post‐ICU support particularly described reminders and follow‐up contact as important for sustaining engagement: ‘The support [text messages] has been motivating… Without the reminders, it would probably have been left on the shelf’ (P4). However, due to the limited number of patients within each support condition, these findings should be interpreted as qualitative feasibility signals rather than evidence of comparative differences between groups.

Some patients described continued use beyond the study period, often through alternative cognitive activities such as mobile applications: ‘Now I have at least five apps at a time’ (P2), reflecting sustained engagement extending beyond the original intervention purposes. Written materials delivered as part of the two interventions were generally perceived as clear, whereas recall of verbal information provided during ICU admission varied.

Nurses reported similar variability in delivery. Although some described the intervention components as largely used as intended, others reported inconsistent delivery, influenced by competing clinical priorities and patient readiness: ‘I believe we used it 50/50 as intended’ (N5). Nurses generally felt adequately prepared to deliver the interventions and described the materials as accessible and easy to use, although they sometimes forgot to use them: ‘The main challenge was remembering to deliver it rather than the time required’ (N4).

Brain Training activities were described as easier to introduce, whereas Mindfulness occasionally required more explanation depending on patient tolerance. Nurses emphasised maintaining voluntariness and respecting refusals to avoid pressuring patients: ‘If they declined, this was respected and documented without further insistence’ (N5).

Overall, adherence varied across patients and over time, reflecting flexible and adaptive use influenced by patient capacity, contextual factors and available support, rather than standardised implementation.

##### 5.2.2.2 Dose

Patients reported variation in frequency and duration over the recovery trajectory, often adjusting use according to daily energy levels and competing demands. Brain Training was typically used one to three times per week for 15–30 min, although some described more frequent use early after discharge. Engagement was often adjusted in response to other rehabilitation activities and resumption of daily responsibilities: ‘It has varied, but on average it has been less than an hour per week’ (P8). Mindfulness was commonly used two to four times per week, with shorter audio files preferred: ‘It's much easier to say, “I'll take one now,” if it's no more than ten minutes’ (P4). Several patients described adjusting frequency and duration pragmatically according to daily energy levels, sometimes integrating exercises into existing routines or substituting with alternative activities.

Nurses described daily delivery during ICU admission as difficult to sustain due to workload, shift patterns, competing clinical tasks and variation in patient readiness: ‘Daily use was difficult to maintain’ (N1). Despite this, some emphasised the importance of integrating cognitive rehabilitation into routine care. ‘Just as we routinely do physical care and mobilisation, cognitive rehabilitation should be embedded into everyday practice’ (N4).

Overall, dose was affected by patient energy levels, competing demands, clinical context and daily routines, rather than predefined targets.

## 6 Discussion

This multi‐method feasibility evaluation explored the acceptability and fidelity of two cognitive rehabilitation components, Mindfulness and Brain Training, delivered from ICU admission to 6 months after discharge. Overall, both intervention components were perceived as acceptable and relevant by patients and nurses. However, fidelity varied across recovery phases and care settings, and high acceptability did not consistently translate into sustained delivery or engagement. This variability was closely linked to patient readiness and contextual conditions, rather than reflecting rejection of the intervention itself.

*Acceptability* appeared context‐dependent and varied across recovery phases and patient conditions. During ICU admission, our findings align with research on early mobilisation, demonstrating that participation in supportive interventions during critical illness is closely linked to physiological stability, staffing resources and organisational capacity [28, 29]. These findings suggest that perceived burden during ICU admission was primarily related to limited cognitive readiness and organisational capacity, rather than appraisal of the intervention itself.

After ICU discharge, our findings on variable engagement align with most post‐ICU follow‐up research showing that, although patients value structured support interventions, maintaining sustained participation is challenging once hospital‐based care is reduced and recovery becomes increasingly self‐managed [30, 31, 32]. Cox et al. similarly observed that mindfulness‐based rehabilitation was generally acceptable to ICU survivors, but engagement varied according to readiness, symptom burden and perceived personal fit [33]. These findings indicate that acceptability in this context is not static but influenced by patients' capacity to engage and competing demands across the different stages of recovery. Importantly, these context‐dependent patterns of acceptability did not consistently translate into sustained delivery or adherence.

During ICU admission, our findings on *fidelity* are consistent with known barriers in ICU rehabilitation research, where delivery of supportive interventions is influenced by organisational capacity, staffing constraints and competing care priorities [29, 34]. Similarly, Wassenaar et al. reported that cognitive exercises during ICU admission were feasible primarily in clinically stable patients and required flexibility to accommodate fluctuations in attention and tolerance [35]. In the context of the present study, fidelity was characterised by variable adherence and reduced dose, reflecting challenges in initiating and sustaining delivery within routine care rather than lack of acceptance. Differences between the two intervention components further illustrate how fidelity was affected in this phase. Brain Training, which required sustained cognitive effort, was more sensitive to fluctuations in attention and energy, whereas shorter Mindfulness exercises were more deliverable during ICU admission. This pattern is consistent with previous ICU‐based studies demonstrating that participation in cognitive activities depends on alertness, tolerance and perceived effort [35, 36].

After ICU discharge, our findings indicate that variability in fidelity was increasingly influenced by the transition to self‐management, where responsibility for engagement shifted from ICU nurses to patients and their available support. Although the study was not designed to compare intervention groups formally, patients receiving structured post‐ICU support appeared more likely to describe reminders and follow‐up contact as helpful for sustaining intervention use across recovery phases. These findings suggest that continuity of support may be important for maintaining engagement after hospital discharge and warrant further investigation in future studies. In line with post‐hospital rehabilitation research, the transition from the nurse‐supported delivery in the ICU to self‐managed use after ICU discharge appeared to influence sustained use across recovery phases, emphasising the importance of continued support beyond the hospital setting, particularly the role of relatives in sustaining engagement [37, 38].

The observed decline in consistency of use after discharge may therefore reflect reduced continuity of structured support, rather than reduced perceived relevance. Across phases, fidelity was characterised by flexible and selective use aligned with patient capacity, supporting perspectives from complex intervention research, where adaptation is considered a pragmatic and necessary response to heterogeneous needs and dynamic recovery trajectories rather than a simple protocol deviation [16, 37, 39].

Overall, fidelity in this study is best understood as reflecting how intervention use was adapted to patients' capacity across recovery phases, rather than strict adherence to a predefined protocol. This suggests that future evaluations may benefit from focusing less on uniform delivery targets and defining acceptable ranges of use and dose, alongside improved integration into routine care and support for self‐management after discharge.

**FIGURE 1:**
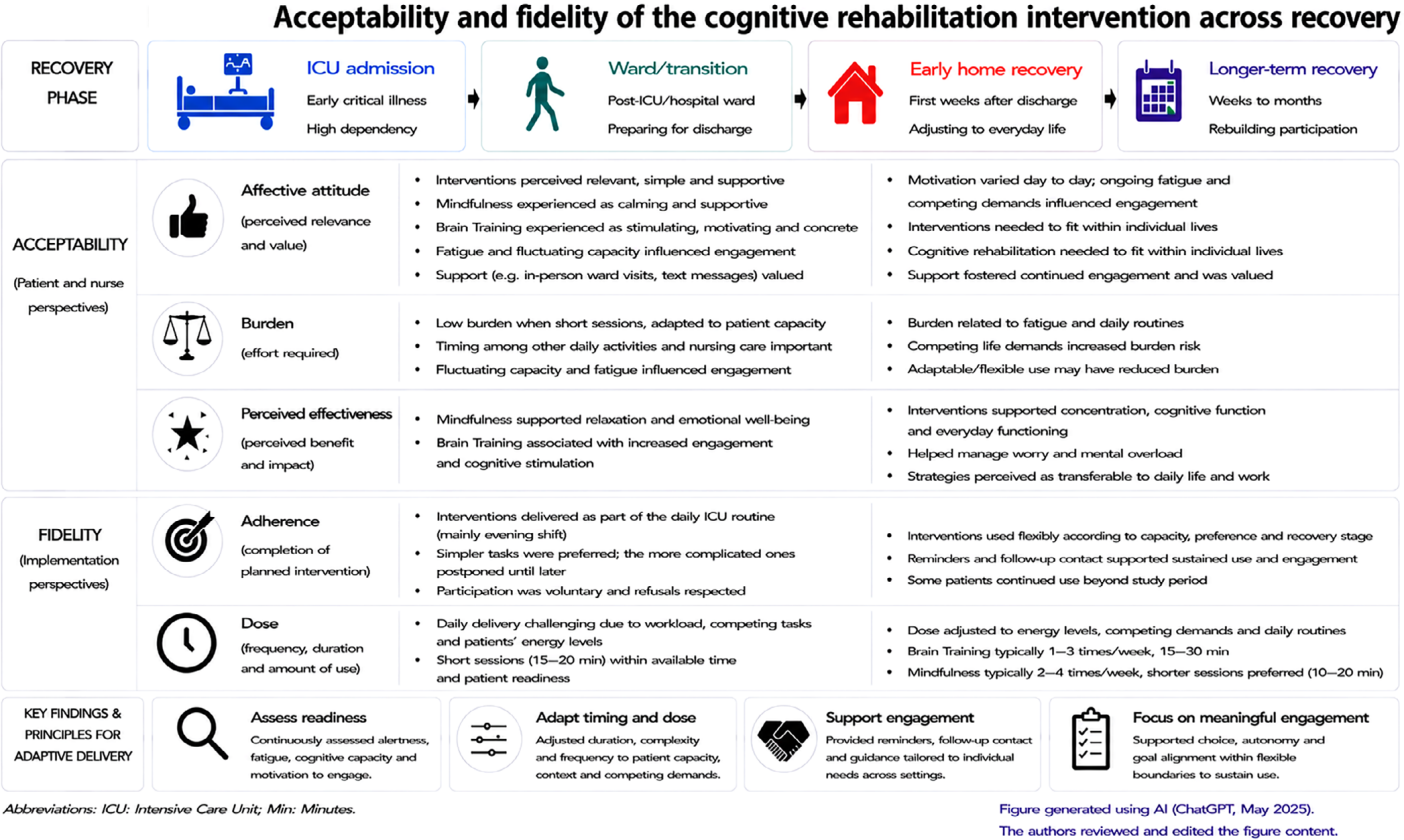
Acceptability and fidelity of the cognitive rehabilitation intervention (Mindfulness or Brain Training) across recovery phases after critical illness. The figure integrates findings related to engagement, perceived burden, adherence and dose across the ICU‐to‐home recovery trajectory. Intervention use appeared to vary according to patient capacity, fatigue, competing demands, contextual conditions and available support and was characterised by flexibility and adaptation rather than standardised delivery. Activities, timing, frequency and support were adjusted according to recovery stage and individual needs.

Figure 1 illustrates how acceptability and fidelity of the cognitive rehabilitation intervention appeared to vary across recovery phases after critical illness. The figure integrates findings related to engagement, perceived burden, adherence and dose across the ICU‐to‐home recovery trajectory, highlighting how intervention use required ongoing adaptation according to patient capacity, fatigue, competing demands, contextual conditions and available support.

### 6.1 Strengths and Limitations

Several methodological *strengths* warrant consideration. A key strength was the multi‐site design spanning ICU admission and post‐discharge phases, enabling evaluation of how intervention delivery and use varied across clinical contexts, recovery stages and levels of available support. This included the transition from nurse‐supported delivery in the ICU to patient‐led self‐management after discharge, a phase where reduced support appeared to influence sustained engagement and consistency of use.

The combination of quantitative and qualitative data provided complementary insights into how the intervention was perceived and used, linking reported relevance and acceptability to actual patterns of delivery and engagement in practice. By examining adherence alongside participant responsiveness and quality of delivery, the study provided a more nuanced understanding of fidelity beyond frequency and duration alone.

Several *limitations* should be considered. Delivery was influenced by contextual conditions inherent to intensive care, including sedation, delirium and fluctuating cognition, which may have limited patient engagement during ICU admission and continuation after ICU discharge. Nurses were central to delivery, and their ability to support the intervention depended on workload and competing clinical priorities. Organisational and cultural variation across participating ICUs may also have contributed to differences in delivery patterns, potentially affecting consistency in adherence and dose. However, the study was not designed to evaluate site‐specific differences formally.

At the same time, these factors reflect the real‐world conditions under which the intervention is intended to be delivered. As such, the observed variability provides a pragmatic representation of implementation in routine clinical practice, strengthening the validity and relevance of the findings for future application.

The survey instruments included a limited number of items, which may have restricted the breadth of quantitative assessment. Surveys were intentionally kept brief to minimise burden and support completion during ICU admission and longitudinal follow‐up. While this may have constrained measurement detail, it supported feasibility and alignment across ICU and post‐discharge data collection.

Nevertheless, the evaluation sample was still limited, and only a proportion of enrolled patients contributed to the repeated survey data, reducing the robustness of quantitative estimates. Likewise, variability in completion of nurse registrations reduced the precision of fidelity assessment during ICU admission. In addition, data were primarily obtained from patients who engaged with the intervention, and perspectives from those who did not engage or did not complete follow‐up were not explored in depth, introducing potential selection bias.

Finally, as an early‐phase evaluation of intervention delivery, the findings reflect exploratory implementation under real‐world conditions and may not be generalisable beyond similar healthcare contexts at this stage.

## 7 Implications and Further Research

The results from this study suggest that future work should focus on how cognitive rehabilitation is delivered, rather than on substantial modification of intervention content. In particular, delivery approaches that allow flexibility in timing and intensity and that align with patients' cognitive readiness and energy levels may be important to support engagement across recovery phases.

Integration into routine care processes, including clearer roles for staff and local ownership of delivery, may support more consistent delivery during ICU admission. In addition, continuity of support across the ICU‐to‐home transition appears important, as reduced support after discharge was associated with less consistent use. Approaches to support self‐management, including involvement of relatives or follow‐up contact, warrant further investigation. Future evaluations may also benefit from defining acceptable ranges of intervention use and dose that reflect variation in patient capacity and recovery trajectories, rather than relying on fixed delivery targets.

## 8 Conclusion

The ICU CogHab intervention components were perceived as relevant and meaningful by both patients and ICU nurses, supporting the feasibility of addressing cognitive recovery early in the critical care trajectory. However, delivery and sustained engagement varied across recovery phases and care settings and were influenced by interacting patient‐ and system‐related factors, including cognitive readiness, fatigue and competing demands.

These findings indicate that, while the intervention components were overall acceptable, consistent delivery depends on alignment with patient capacity and adequate support across the ICU‐to‐home transition before progression to larger‐scale evaluation.

## Author Contributions

K.A. conceived and designed the study, coordinated data collection, conducted the analyses and drafted the manuscript. A.H., P.D., H.K.N. and N.R. contributed to study design and provided methodological and clinical supervision. K.A. led data collection and analysis with support from A.H. and P.D. All authors contributed to the interpretation of the findings and manuscript revision, approved the final version and accept accountability for the work.

## Funding

This study was supported by the Novo Nordisk Foundation (NNF20OC0061394) and Aarhus University. The funders had no role in the study design, data collection, analysis or manuscript preparation.

## Ethics Statement

The study was conducted in accordance with the Declaration of Helsinki and approved by the local Research Ethics Committee of the Central Denmark Region (record no. 1‐10‐72‐137‐23).

## Consent

Written informed consent was obtained from all participants prior to inclusion.

## Conflicts of Interest

The authors declare no conflicts of interest.

## Supporting information


**Supplementary File A** The interview guide used for the ICU nurses.

**Supplementary File B** The interview guide used for patients allocated to Brain training.

**Supplementary File C** The interview guide used for patients allocated to Mindfulness.

## Data Availability

The datasets generated and analysed during the current study are not publicly available due to data protection regulations but are available from the corresponding author upon reasonable request.
