# Supplementary material for: Acceptability and Fidelity of a Cognitive Rehabilitation Intervention During and After Intensive Care: A Feasibility Evaluation
Source: Nurs Crit Care. 2026 Jun 16;31(4):e70544. doi: 10.1111/nicc.70544 (PMC13272925; doi:10.1111/nicc.70544)
Supplement: Supplementary file 1 — Supplementary File A The interview guide used for the ICU nurses. [file NICC-31-0-s003.docx]

| Part 1Introductory questions | **Interview questions** | **Follow-up questions /Probes** |
| --- | --- | --- |
| Experience with the intervention **Materials and usability**  **Information and preparation** | *To begin with, could you describe how much experience you have had using the mindfulness and/or brain training boxes with patients?*  *What has been your overall experience of using mindfulness and/or brain training activities with patients in the ICU?*  *-----------------------------------*  *How did you experience the materials (e.g. activity book, ball, puzzles, calendar, mindfulness leaflet, information pamphlets) when using them in ICU practice?*  *--- --- -------------------------------------------------------*  *How did you experience the information or preparation you received about using the mindfulness and brain training activities with patients?*  *--- --- -----------------------------------------------------*   - *Did you read the two pamphlets included in the boxes?* | **Could you tell me more about that? When you say …, do you mean that …? Could you explain why or why not?**  ------------------------------------  Probes:   \|  \| - Was there anything about the materials that did not work particularly well? - Was there anything that worked especially well? \| \| \| --- \| --- \| --- \| \|  \| - Were there elements of the boxes that you used particularly often with patients? \| \| \|  \| - Were there elements that you did not use at all? If so, why? \| \|  \| - How did you experience the levels of difficulty or complexity of the exercises? \|   ---------------------------------  Probes:   - Was the information or instruction sufficient? - Did you feel that you needed additional support in how to use the mindfulness or brain training activities with patients?   ----------------------------------------   - If yes, how were they to read? - If no, why not? |

| Part 2 – Research Questions | | |
| --- | --- | --- |
| Acceptability (TFA) | **Interview questions** | **Follow-up questions /Probes** |
| Affective attitude | ***Do you feel it made sense for you as a nurse to use mindfulness and/or brain training with ICU patients?*** | Probes:   - Can you describe a positive experience with the brain training? - Can you describe a less positive experience? |
| Burden | ***How did using brain training and/or mindfulness fit into your daily work?***  ***Were there situations where it felt particularly difficult or overwhelming?*** | Probes:   - In terms of time, workload, the patient’s energy level, or your own competencies - Did this vary during the period in which the intervention was tested? |
| Perceived effectiveness | ***To what extent did you experience that mindfulness and/or brain training could have a positive influence on patients’ recovery?***  ***How optimistic are you that mindfulness and brain training may help prevent or reduce cognitive difficulties after critical illness?*** | Probes:   - Please feel free to provide examples if you have any. |

| Fidelity | **Interview question** | **Follow-up questions /Probes** |
| --- | --- | --- |
| AdherenceQuality of intervention delivery | ***Did you feel that you were able to use the mindfulness and/or brain training activities as intended?***  ***How did you feel about being the nurse who introduced and used mindfulness and brain training with the patients (when relevant together with relatives)?*** | Probes:   - What made it easier or more difficult to use them? (e.g. organisation of work, collaboration, your own motivation, patient responsiveness) - What typically influenced whether the activities were used more or less frequently?   For example:   - Organisational factors (time, support, interdisciplinary collaboration, ICU culture) - Personal motivation or competencies - Patients’ or relatives’ responses to the activities |
| Dose | ***We recommended brief daily use of the boxes when possible. How did this work in practice?*** | Probes:   - Approximately how much time was typically spent using the activities? - Did you feel that the amount (dose) was appropriate? - What would be necessary to make it feasible to use mindfulness or brain training daily (for example in the same way as mobilisation is incorporated into daily care)? |
| Closing questions | \| What helped facilitate the use of mindfulness or brain training in practice? \| \| --- \|  \| What barriers did you experience? (e.g. time, organisational support, patient motivation, equipment or resources) \| \| --- \|  \| How was your overall experience of participating in the project? \| \| --- \|  \| What would you have liked to be different in the intervention, or if the intervention were to be implemented in the future? \| \| --- \|  \| Is there anything important that I have not asked about but that you think should be included? \| \| --- \|  \| Do you have any questions or anything you would like clarified? \| \| --- \| |  |
