# Supplementary material for: Acceptability and Fidelity of a Cognitive Rehabilitation Intervention During and After Intensive Care: A Feasibility Evaluation
Source: Nurs Crit Care. 2026 Jun 16;31(4):e70544. doi: 10.1111/nicc.70544 (PMC13272925; doi:10.1111/nicc.70544)
Supplement: Supplementary file 3 — Supplementary File C The interview guide used for patients allocated to Mindfulness. [file NICC-31-0-s001.docx]

| Part 1Introductory questions | **Interview questions** | **Follow-up questions /Probes** |
| --- | --- | --- |
| General recovery | ***How are you doing today, now that it has been about six months since your ICU admission?*** | **Could you tell me more about that? When you say …, do you mean that …? Could you explain why or why not?** |
| Introduction to the Mindfulness box **Information and guidance** | **Can you tell me what you thought about the Mindfulness exercises (the audio-files)?**  *Probes:*   - *What did you think about the overall design of the materials?* - *Were there any audio recordings that you used particularly often?* - *Were there any audio recordings that you did not use at all?* - *What did you think about the length of the audio recordings?*   ***How well did you feel informed or guided in how to use the Mindfulness exercises?***  *--- --- ---*   - *Did you read the two pamphlets included in the box?* - If yes, how were they to read? - If no, why not? | Probes:   - Why were these exercises relevant (or not relevant) for you? - Was there anything you liked less? - How did you experience the level of difficulty or complexity of the exercises?   Probes:   - Did you read the intervention information included in the box? - Was the information sufficient to help you use the exercises? - How easy or difficult were the materials to understand? |
| Part 2 – Research questions | | |
| Acceptability (TFA) | **Interview questions** | **Follow-up questions /Probes** |
| Affective attitude | ***How relevant was Mindfulness for you?***  ***Did it make sense for you to use the Mindfulness exercises?*** | Probes:   - Can you describe a positive experience with Mindfulness? - Can you describe a less positive experience? |
| Burden | ***How was your energy and mental capacity when using the Mindfulness exercises?*** | Probe:   - Did this change from your ICU stay until now? |
| Perceived effectiveness | ***What impact, if any, has Mindfulness had on your recovery after intensive care?***  ***How optimistic are you about the potential benefits of Mindfulness?*** | Probe:   - Do you feel that the Mindfulness exercises made a difference for you? - Do you feel the exercises helped with possible cognitive difficulties such as memory, overview, attention, concentration, or problem solving in everyday activities? |

| Fidelity | **Interview questions** | **Follow-up questions /Probes** |
| --- | --- | --- |
| Adherence | ***Do you remember receiving help with the Mindfulness exercises while you were in the ICU?***  ***How did you use the Mindfulness exercises later on the ward?***   - - ***and after discharge?***   *----------*  ***How did you feel about being responsible for using the exercises yourself after ICU discharge (possibly with support from relatives)?*** | Probes:   - Did your use of the exercises vary over time from ICU admission until now? - Was anything challenging about using the exercises? - What usually influenced whether you used the Mindfulness exercises or not?   ------------   - Did you feel that you lacked support when using the Mindfulness exercises after ICU discharge? |
| *Additional question for the “support post-ICU” group* | ***Did the support and reminders you received from me during the project period make a difference for you?*** | Probe:   - For example the follow-up visits on the ward after transfer or the text messages and phone calls you received. |
| Dose | ***How much did you use the Mindfulness exercises overall?*** | Probe:   - What do you think is important in order to use Mindfulness daily/regularly (as recommended)? |
| Closing questions | **Can you describe what it has been like to participate in this project?**  **Is there anything you would have liked to be different?**  **Is there anything else about the Mindfulness exercises that you would like to tell me?**  **Do you have any questions or anything you would like clarified?** |  |
